# Flight safety assessment based on an integrated human reliability quantification approach

**DOI:** 10.1371/journal.pone.0231391

**Published:** 2020-04-16

**Authors:** Yundong Guo, Youchao Sun

**Affiliations:** College of Civil Aviation, Nanjing University of Aeronautics and Astronautics, Nanjing, PR China; Tongii University, CHINA

## Abstract

Human error is an important risk factor for flight safety. Although the human error assessment and reduction technique (HEART) is an available tool for human reliability derivation, it has not been applied in flight safety assessment. The traditional HEART suffers from imprecise calculation of the assessed proportion of affect (APOA) because it heavily depends on a single expert’s judgment. It also fails to provide remedial measures for flight safety problems. To overcome these defects of the HEART, this study proposes an integrated human error quantification approach that uses the improved analytic hierarchy process method to determine the APOA values. Then, these values are fused to the HEART method to derive the human error probability. A certain flight task is completed to assess human reliability. The results demonstrate that the proposed method is a reasonable and feasible tool for quantifying human error probability and assessing flight safety in the aircraft manipulation process. In addition, the critical error-producing conditions influencing flight safety are identified, and improvement measures for high-error-rate operations are provided. The proposed method is useful for reducing the possibility of human error and enhancing flight safety levels in aircraft operation processes.

## Introduction

Computer technology has been widely used in aviation industries. The integration of airborne electronic equipment has made aircraft manipulation more automatic and intelligent, which has greatly improved the reliability and safety of aircraft [1, 2]. However, accurate manipulation by the flight crew is still critical during the flight process. While the proportion of flight accidents caused by mechanical factors has decreased dramatically, the proportion of flight accidents caused by human factors has gradually increased [3, 4]. The position of the flight crew in a large-scale flight automation system has not been well studied despite its role in many flight accidents and accident symptoms. It is estimated that more than 60% of flight accidents are caused directly or indirectly by human error [5, 6]. In addition, more than 90% of nuclear power plant accidents [7, 8], more than 80% of petrochemical industry accidents [9, 10], and more than 75% of maritime accidents [11, 12] are related to human actions. Therefore, human factors should be considered in the process of accident analysis to ensure the effective prevention of risk events [13, 14].

Swain defined human reliability as the ability to perform a given task without errors in a definite time and under certain requirements [15]. Human reliability analysis (HRA) consists of three main phases: identifying human behaviors, establishing a model of critical human behaviors, and determining the probability of human error. Although researchers have proposed many models for predicting the probability of human error, these approaches contain many defects, such as a lack of data, subjectivity and uncertainty of analysis [16]. Hence, HRA techniques should be improved to solve these defects.

Numerous HRA approaches have been proposed to calculate human error probability (HEP) and evaluate human performance. The technique for human error rate prediction (THERP) is one of the typical HRA methods [17]. It aims to calculate the HEP of necessary actions based on task event tree analysis. In its original use, THERP relied heavily on nuclear power plant data and experts’ judgments and was popular and useful for HEP assessment in the nuclear field. However, the scenario and characteristics of aviation tasks are different, and therefore, the THERP is difficult to apply directly to assess pilots’ HEP. Success likelihood index methodology (SLIM) is a decision-analytic technique that depends primarily on the quantification of experts’ preferences and judgments. This technique uses performance-shaping factors to derive a success likelihood index (a type of preference index), and then, the index is calibrated with existing data to calculate the final HEP. Although SLIM has been applied in maritime transportation, coal mines, and other areas [18, 19], it relies on experts’ subjective determination of performance-shaping factors, which reduces the objectivity and accuracy of analytical results. The human factor analysis classification system is an error taxonomic method for aviation accident analysis. It assumes that human errors are caused by the failure of four core system layers: organizational influences, unsafe supervision, preconditions for unsafe acts and unsafe acts [20]. Although this approach can be used to qualitatively analyze accident causality in accident scenarios, it can only identify human errors rather than quantify the HEP. Cognitive reliability and error analysis has been considered a profound reform of qualitative HRA, and new approaches to cognitive error processing have been derived from the diversification of error models. The contextual control model is a cognitive model based on four cognitive functions: observation, interpretation, planning and execution [21, 22]. According to this model, cognitive function errors are the root causes of human errors. The cognitive reliability and error analysis method assumes that there are four observational errors, also known as error modes: scrambled, opportunistic, tactical, and strategic control modes. Nevertheless, the quantification process of this method is complex, and it offers no remedial measures for human errors. The human error assessment and reduction technique (HEART) summarizes eight general categories to classify operator tasks and employs highly structured error-producing conditions (EPCs), which are derived from the mass ergonomics literature as well as accident data from various fields such as aviation, nuclear power production and maritime transportation [23]. The HEART addresses only human errors that have a significant influence on the system, thereby reducing resource usage. However, its validation and rationality in terms of flight safety assessment remain uncertain. Furthermore, the quantitative process of the HEP in this technique is subjective, which reduces the consistency and reliability of the obtained results.

Based on the original HEART, improved methods have been proposed in different fields [19]. Chadwick [24] used a participative team approach to reduce the subjectivity of HEP, and a case study in a critical nursing task showed that this method successfully ranks the related EPCs that influence human errors. However, the disadvantage of this approach is that the EPC weights are still subjectively graded by participative team experts. Akyuz et al. [25] introduced an analytic hierarchy process to derive the assessed proportion of EPCs. This method improves the quantification of expert judgment to some extent and obtains satisfactory practical outcomes in maritime HEP assessment. The drawback of this approach, however, is that a consistency test of the judgment matrix is always necessary. If the consistency test fails, a new judgment matrix needs to be constructed. In addition, this method is time-consuming. Akyuz et al. [26] employed interval type-2 fuzzy sets to cope with the uncertainty of experts’ judgment on the assessed proportion of EPCs during cargo operation, in which reliable and available HEP calculations are performed. However, the calculation process is very complicated. Kumar et al. [27] employed a triangular fuzzy function to determine the EPC weights in a nuclear power plant. However, this approach enables the HEART technique to accommodate experts’ judgments with uncertainty. Furthermore, it is necessary to ensure that experts’ experience is rich enough to construct fuzzy rules, and the calculation is very complex. In addition, all of the aforementioned improved HEART methods cannot be fully applied to flight safety assessment, considering that flight task scenarios and intensive operating tasks are substantially different from those in nuclear, maritime, healthcare, and other fields.

To objectively assess flight safety based on human reliability values of the aircraft control process, this study develops an integrated method to quantify HEP. This approach considers the influence of organization and management factors in the aircraft manipulation process and employs the improved analytic hierarchy process (IAHP) method to determine the weight of EPCs, which can be used to precisely derive the HEP. The results of this work could provide safety recommendations and suggestions for flight crews to improve flight safety.

## Methods

### HEART technique

The core of the HEART method is to study EPCs that have a negative impact on human performance and to seek remedial measures for reducing HEP. The HEART has been successfully applied in many fields, such as nuclear power plants, marine and offshore operations, and radiotherapy treatment [28].

The HEART method consists of three primary parameters: nominal human error probability (NHEP), strength of EPCs and weight of EPCs. The NHEP value can be determined by the corresponding task type. Generally, there are eight kinds of generic tasks, from A to H [29]. If the description of the task process for HRA does not accord with these eight task types, safety engineers or analysts should consider selecting the M-type task. EPCs are defined as human performance-shaping factors during the implementation procedure that affect the HEP value connected with a generic task. HEP is always directly influenced by these EPCs in a specific task, according to the HEART methodology. Through statistical analysis Williams obtained the NHEP and strength of EPC values, which were derived from various fields, such as nuclear plants, maritime affairs, and chemical liquid tankers [29]. The method starts with selecting generic task types based on specific task processes. Afterwards, the corresponding NHEP value is determined. Then, the corresponding strength values of EPCs are selected from 38 descriptions. If there are many EPCs in a specific task, the APOA needs to be determined by a single expert. In these circumstances, the final HEP can be calculated using Eq (1) [30].

$$HEP=NHEP\times \left\{\prod_{i}\left[\left(EPC_{i}-1\right)\times APOA_{i}+1\right]\right\},(\text{i}=1,2,3,\cdots ,38)$$ (1)

In this equation, *EPC*_*i*_ indicates the *i*th EPC, and *APOA*_*i*_ is the *i*th assessed proportion of affect, which is the weight value of the *i*th EPC.

### IAHP method

In the traditional analytic hierarchy process, there are uncertainties in the judgment matrix for calculating weights. The fuzzy analytic hierarchy process is an improvement of the traditional AHP; it is mainly used in economics and enterprise management and rarely used in human reliability assessment [31, 32]. However, the calculation results based on the fuzzy analytic hierarchy process method are less accurate. To optimize the computational process and ensure accuracy, this study introduces the IAHP to quantify human reliability in aviation risk assessment. This method uses a 0.1–0.9 scale, which motivates experts or safety engineers to make decisions regarding the relative importance of two factors. In addition, the fuzzy congruous matrix transformed by the priority matrix satisfies the consistency condition, and it is not necessary to perform a consistency test. Furthermore, the method can greatly reduce iterations, improve the convergence speed, and satisfy the requirements of calculation accuracy. The following are the specific implementation steps of the IAHP:

1. Establish a judgment matrix *A* = (*a*_*ij*_)_*n*×*n*_ that can describe the relative importance of each factor according to the hierarchical structure of the evaluation object. Considering the difference in human cognition and the complexity of objective things, it is first necessary to select more than one expert to construct judgment matrix $A^{\left(k\right)}={\left(\bar{a_{ij}}\right)}^{\left(k\right)}{}_{n\times n}$ separately. Then, an integrated judgment matrix is built by the credit degree method of group decision. The final result for matrix *A* = (*a*_*ij*_)_*n*×*n*_ can be derived with Eqs (2)–(4) [33, 34].
  $$A^{\left(k\right)}={\left(\bar{a_{ij}}\right)}^{\left(k\right)}{}_{n\times n}=\left[\begin{array}{cccc} \bar{a_{11}} & \bar{a_{12}} & \cdots & \bar{a_{1n}}\\ \bar{a_{21}} & \bar{a_{22}} & \cdots & \bar{a_{2n}}\\ \vdots & \vdots & \vdots & \vdots \\ \bar{a_{n1}} & \bar{a_{n2}} & \cdots & \bar{a_{nn}} \end{array}\right]$$ (2)
  $$T(k)=\frac{\psi_{k}}{\sum_{q=1}^{m}\psi_{q}}$$ (3)
  $$a_{ij}=\sum_{k=1}^{m}\bar{a_{ij}}\times T(k)$$ (4)
  where *A*^(*k*)^ is the judgment matrix constructed by the *k*-th expert and *T*(*k*) is the weight of experts. Assuming that the participating experts are classified into different categories based on their experience and knowledge, the identified category including the *k*-th expert has *ψ*_*k*_ experts. The weight of the *k*-th expert can be expressed with Eq (3) [34]. *A* is the fuzzy reciprocal judgment matrix, and $\bar{a_{ij}}\in \left[0.1,0.9\right]$. If $\bar{a_{ii}}=0.5$, it means that factor $\bar{a_{i}}$ is as important as $\bar{a_{j}}$; if $\bar{a_{ij}}\in \left[0.1,0.5\right)$, it means that $\bar{a_{j}}$ is more important than $\bar{a_{i}}$; and if $\bar{a_{ij}}\in \left(0.5,0.9\right]$, it means that $\bar{a_{i}}$ is more important than $\bar{a_{j}}$. The criteria of the fuzzy judgment matrix are shown in Table 1 [33].
2. Convert matrix A into fuzzy congruous matrix *R* using Eqs (6) and (7) [35]. In this context, matrix *R* satisfies the consistency condition, which can simplify the computational process.
  $$R={\left(r_{ij}\right)}_{n\times n}=\left[\begin{array}{cccc} r_{11} & r_{12} & \cdots & r_{1n}\\ r_{21} & r_{22} & \cdots & r_{2n}\\ \vdots & \vdots & \vdots & \vdots \\ r_{n1} & r_{n2} & \cdots & r_{nn} \end{array}\right]$$ (5)
  $$r_{i}=\sum_{j=1}^{n}a_{ij}$$ (6)
  $$r_{ij}=\frac{r_{i}-r_{j}}{2n}+0.5$$ (7)
3. Calculate the relevant weight of each EPC. Weight vector *w* can be calculated by the normalization method, sorting method or square root method [36]. In this paper, the square root method is selected to ensure high accuracy. Therefore, the weight vector is expressed as
  $$w={\left[w_{1},w_{2},\cdots ,w_{n}\right]}^{\text{T}}={\left[\frac{\sqrt[{n}]{\prod_{j=1}^{n}r_{1j}}}{\sum_{i=1}^{n}\sqrt[{n}]{\prod_{j=1}^{n}r_{ij}}},\frac{\sqrt[{n}]{\prod_{j=1}^{n}r_{2j}}}{\sum_{i=1}^{n}\sqrt[{n}]{\prod_{j=1}^{n}r_{ij}}},\cdots ,\frac{\sqrt[{n}]{\prod_{j=1}^{n}r_{nj}}}{\sum_{i=1}^{n}\sqrt[{n}]{\prod_{j=1}^{n}r_{ij}}}\right]}^{\text{T}}$$ (8)

**Table 1:** Criteria of fuzzy judgment matrix.

Value	Definition	Description
0.5	Equally important	Equally important compared with the other
0.6	Slightly important	One factor is slightly more important than the other
0.7	Obviously important	One factor is obviously more important than the other
0.8	Much more important	One factor is much more important than the other
0.9	Extremely important	One factor is extremely more important than the other
0.1, 0.2, 0.3, 0.4	Converse comparison	If $\bar{a_{i}}$ is *p*_*ij*_ compared with $\bar{a_{j}}$, then $\bar{a_{j}}$ is *p*_*ji*_ = 1 − *p*_*ij*_ compared with $\bar{a_{i}}$

### Integrated human reliability quantification approach

**Fig 1:**
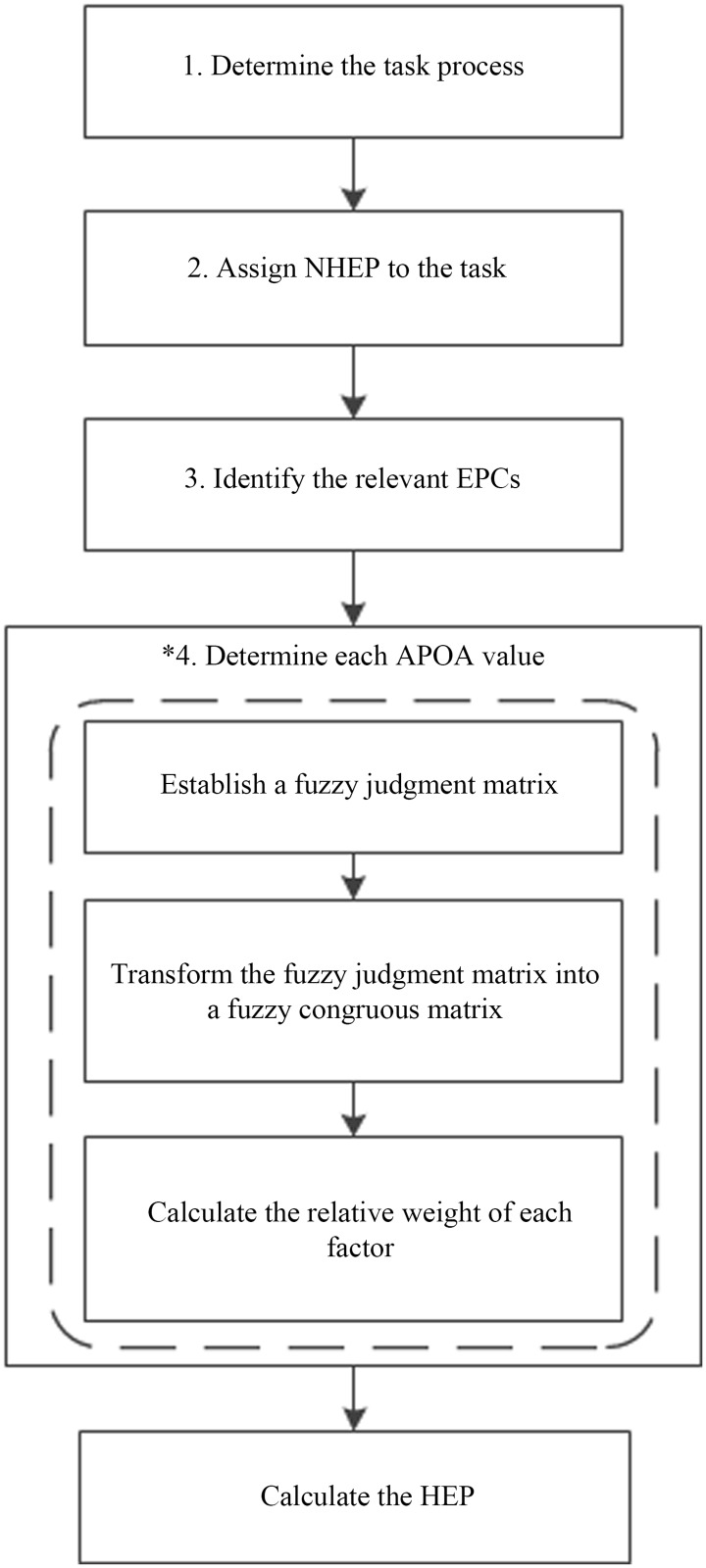
The flow chart of the proposed method.

In this section, the integrated IAHP-HEART method is proposed to quantify human reliability for assessing flight safety. A flow chart of the proposed method is shown in Fig 1, and the its specific implementation steps are as follows.

Step 1: Determine the task process or events based on specific task scenarios. Then, perform a comprehensive list of event sequences by hierarchical task analysis for further study, where the main steps are broken up into a series of subtasks or elementary actions [21].
Step 2: Identify the generic task type for each subtask based on the analysis results. Then, the corresponding NHEP value of each subtask can be determined.
Step 3: Select the EPCs associated with each subtask from the 38 EPCs that have a negative impact on human operational reliability and may increase the probability of human error.
*Step 4: The subjective judgment of a single expert determines the APOA values of EPCs based on the original HEART; these APOA values are the proportion of the effect relative to itself. It cannot represent the relative importance between related EPCs. The IAHP approach is introduced to determine the APOA value of each EPC, namely, the weight of the EPC. Moreover, the fuzzy congruous matrix in this method ensures the consistency of the judgment matrix without checking repeatability [37]. It can largely reduce the subjectivity and uncertainty of experts.
  In this context, a fuzzy judgment matrix *A* that can describe the relative importance of each EPC for a specific subtask is established using Eqs (2)–(4). Thereafter, fuzzy judgment matrix *A* is transformed into fuzzy congruous matrix *R* in accordance with Eqs (5)–(7). Then, the relevant weight of each EPC is finally determined with Eq (8), which is the APOA value.
Step 5: After all parameters are determined, apply Eq (1) to calculate the HEP value of each subtask.

## Case study

### Flight task analysis

Flight safety is the most important objective in civil aviation passenger transport. The flight crew requires must simultaneously execute numerous monitor and control activities during the aircraft manipulation process. Therefore, it is essential to quantify human reliability based on flight missions to assess flight safety.

**Fig 2:**
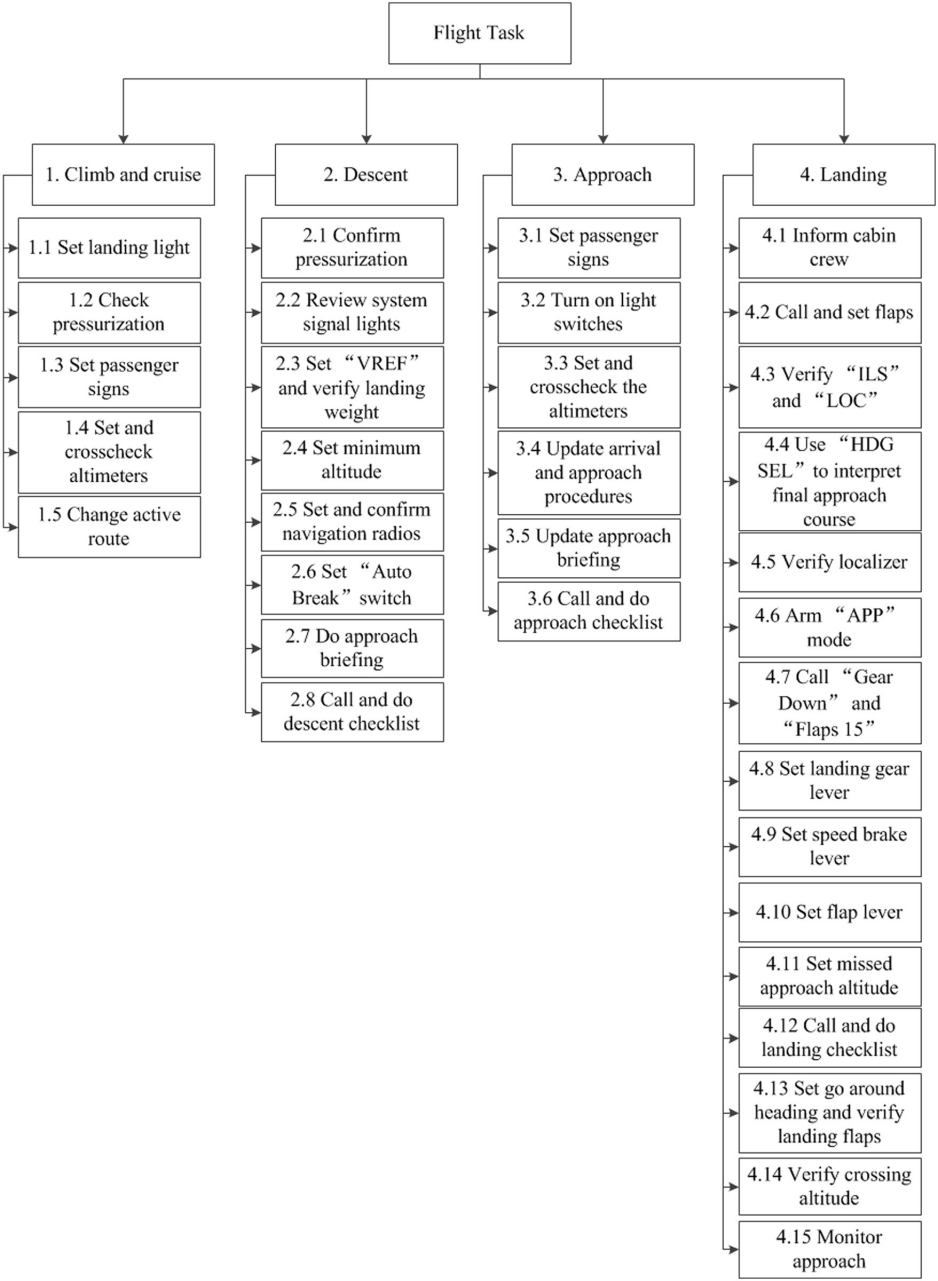
The results of the flight task analysis based on hierarchical task analysis.

By referring to the decomposition of landing missions based on hierarchical task analysis for indigenous defense fighters [38] and considering the standard operation procedure of Boeing 737 [39], a series of flight subtasks are derived based on hierarchical task analysis. Fig 2 presents the analytic results considering the aircraft manipulation process.

Assume that the environment of the flight mission is fine weather. The organization, working conditions, man-machine interface and operational support, availability of procedures, and available time are at a satisfactory level. Flight crew collaboration quality, mental workload, and flight experience are at an acceptable level. Considering that there are many subtasks in HRA, we choose only task 1 as an example.

### Assignment of each subtask NHEP

After obtaining the results of the flight task analysis, the generic task type of each subtask is determined based on task characteristics. Subtask 1.1 is completed by a copilot who has adequate knowledge and experience to assist the captain in controlling the aircraft in flight. Subtasks 1.2 and 1.3 are also implemented by the copilot. The captain and copilot work together to complete subtask 1.4. Subtask 1.5 is performed by the captain, who is primarily responsible for flight safety and issuing flight operation instructions. Clearly, the climb and cruise procedures are completed under the cooperation of the captain and copilot. Based on the above analysis of flight subtask characteristics, the most suitable generic task type can be determined for each subtask. Finally, each subtask NHEP value is derived, as shown in Table 2.

**Table 2:** NHEP and EPCs of each subtask.

Subtask (climb and cruise)	Identified generic task/NHEP	EPCs
1.1 At or above 10000 FT (AGL), set the LANDING light switch to OFF	E/2.0E-2	EPC(23)/(29)/(31)
1.2 Check pressurization, and crosscheck altimeter indication	G/4.0E-4	EPC(1)/(15)/(23)
1.3 Set passenger signs as needed	E/2.0E-2	EPC(1)/(15)
1.4 At transition altitude, set altimeter to standard (STD)	E/2.0E-2	EPC(2)/(15)/(23)/(24)/(32)
1.5 Before top of descent point (T/D), change active route as needed to complete approach	C/1.6E-1	EPC(2)/(17)/(18)/(26)

NHEP, nominal human error probability; EPC, error-producing condition.

### Identification of relevant EPCs

Once the generic task type of each subtask is determined, experts or analysts must identify relevant EPCs for each subtask. Importantly, these relevant EPCs can be checked from the original HEART method, which has 38 EPC descriptions [40]. This is verified in the S1 Table. The EPCs influencing the manipulation process in the climb and cruise tasks are shown in Table 2.

### Determination of APOA value

In this section, the IAHP approach is presented to calculate the APOA value of each EPC. First, four experts construct the fuzzy judgment matrix separately. The four experts classify two categories based on their experience and knowledge. The first category contains three experienced experts, and the second category has one expert with experience and knowledge at general levels. The different judgment matrices based on the four experts are expressed as

$$\begin{array}{cc} A^{\left(1\right)}=\left[\begin{array}{l} \begin{array}{ccccc} 0.5 & 0.\text{4} & 0.\text{6} & 0.6 & 0.7\\ 0.\text{6} & 0.5 & 0.\text{7} & 0.7 & 0.8\\ 0.4 & 0.\text{3} & 0.5 & 0.6 & 0.6\\ 0.4 & 0.3 & 0.4 & 0.5 & 0.\text{4} \end{array}\\ \begin{array}{ccccc} 0.3 & 0.2 & 0.4 & 0.7 & 0.5 \end{array} \end{array}\right] & A^{\left(2\right)}=\left[\begin{array}{l} \begin{array}{ccccc} 0.5 & 0.3 & 0.\text{7} & 0.6 & 0.7\\ 0.7 & 0.5 & 0.8 & 0.\text{6} & 0.8\\ 0.3 & 0.2 & 0.5 & 0.\text{7} & 0.6\\ 0.4 & 0.\text{4} & 0.\text{3} & 0.5 & 0.3 \end{array}\\ \begin{array}{ccccc} 0.3 & 0.2 & 0.4 & 0.7 & 0.5 \end{array} \end{array}\right] \end{array}$$

$$\begin{array}{cc} A^{\left(3\right)}=\left[\begin{array}{l} \begin{array}{ccccc} 0.5 & 0.3 & 0.6 & 0.\text{7} & 0.7\\ 0.7 & 0.5 & 0.8 & 0.7 & 0.8\\ 0.4 & 0.2 & 0.5 & 0.\text{6} & 0.6\\ 0.\text{3} & 0.3 & 0.4 & 0.5 & 0.3 \end{array}\\ \begin{array}{ccccc} 0.3 & 0.2 & 0.4 & 0.7 & 0.5 \end{array} \end{array}\right] & A^{\left(4\right)}=\left[\begin{array}{l} \begin{array}{ccccc} 0.5 & 0.3 & 0.\text{8} & 0.6 & 0.\text{6}\\ 0.7 & 0.5 & 0.8 & 0.7 & 0.8\\ 0.\text{2} & 0.2 & 0.5 & 0.6 & 0.\text{7}\\ 0.4 & 0.3 & 0.4 & 0.5 & 0.\text{4} \end{array}\\ \begin{array}{ccccc} 0.\text{4} & 0.2 & 0.4 & 0.6 & 0.5 \end{array} \end{array}\right] \end{array}$$

According to Eqs (3) and (4), the integrated fuzzy judgment matrix is calculated as

$$T(1)=\frac{3}{3+3+3+1}=0.3;T(2)=0.3;T(3)=0.3;T(4)=0.1$$

$$A=(a_{ij}{)}_{5\times 5}=(\sum_{k=1}^{4}\bar{a_{ij}}\times T(k){)}_{5\times 5}=\left[\begin{array}{l} \begin{array}{ccccc} 0.50 & 0.33 & 0.65 & 0.\text{63} & 0.\text{69}\\ 0.\text{67} & 0.50 & 0.\text{77} & 0.\text{6}7 & 0.80\\ 0.\text{35} & 0.23 & 0.50 & 0.63 & 0.61\\ 0.\text{37} & 0.33 & 0.\text{37} & 0.50 & 0.34 \end{array}\\ \begin{array}{ccccc} 0.31 & 0.20 & 0.\text{39} & 0.66 & 0.50 \end{array} \end{array}\right]$$

Then, matrix *A* is transformed into a fuzzy congruous matrix with Eqs (6) and (7), which can be expressed as

$$R=\left[\begin{array}{l} \begin{array}{ccccc} 0.500 & 0.\text{439} & 0.548 & 0.\text{5}89 & 0.574\\ 0.561 & 0.500 & 0.\text{609} & 0.650 & 0.635\\ 0.452 & 0.311 & 0.500 & 0.541 & 0.526\\ 0.411 & 0.350 & 0.459 & 0.500 & 0.485 \end{array}\\ \begin{array}{ccccc} 0.426 & 0.365 & 0.474 & 0.515 & 0.500 \end{array} \end{array}\right]$$

Finally, the weight of each EPC for subtask 1.4 is calculated with Eq (8). The result is derived as

$$APOA_{S_{1.4}}=w_{S_{1.4}}={\left[w_{1},w_{2},\cdots ,w_{5}\right]}^{\text{T}}={\left[0.2140,0.2389,0.1857,0.1776\text{,}0.1838\right]}^{\text{T}}$$

### Calculation of HEP

After all parameters are obtained, we can calculate the HEP of subtask 1.4 by Eq (1). Thus, the HEP value of subtask 1.4 is

$$\begin{array}{l} HEP_{S_{1.4}}=NHEP_{S_{1.4}}\times \left[\left(EPC_{2}-1\right)\times APOA_{2}+1\right]\times \\ \left[\left(EPC_{15}-1\right)\times APOA_{15}+1\right]\times \left[\left(EPC_{23}-1\right)\times APOA_{23}+1\right]\\ \times \left[\left(EPC_{24}-1\right)\times APOA_{24}+1\right]\times \left[\left(EPC_{32}-1\right)\times APOA_{32}+1\right],\\ =1.77E-2 \end{array}$$

Analogously, the HEP values of other subtasks are determined with the aforementioned computational process. The final results are shown in Table 3. The HEPs of these subtasks calculated by the original HEART are also listed in Table 3.

**Table 3:** Human error probability (HEP) of each flight subtask.

Subtask		Generic task	Error-producing conditions (EPCs)	HEP	
				IAHP-HEART	HEART
1	1.1	E	EPC(23)(29)(31)	1.85E-03	2.31E-1
	1.2	G	EPC(1)(15)(23)	5.33E-03	2.22E-1
	1.3	G	EPC(1)(15)	1.28E-02	3.77E-1
	1.4	F	EPC(2)(15)(23)(24)(32)	1.77E-02	4.85E-1
	1.5	H	EPC(2)(17)(18)(26)	1.75E-04	2.68E-1
2	2.1	G	EPC(13)(17)(24)	1.43E-03	3.88E-1
	2.2	G	EPC(2)(17)	4.99E-03	2.63E-1
	2.3	H	EPC(15)(17)(22)(26)	4.45E-04	4.87E-1
	2.4	G	EPC(1)(15)(22)	2.33E-02	5.19E-1
	2.5	G	EPC(2)(15)(25)(32)	6.93E-04	1.86E-1
	2.6	G	EPC(15)(25)(29)	7.34E-04	1.61E-1
	2.7	G	EPC(14)(25)	2.81E-03	3.95E-1
	2.8	F	EPC(2)(8)(22)(24)	7.92E-02	7.33E-1
3	3.1	G	EPC(1)(15)	2.43E-02	2.82E-1
	3.2	E	EPC(15)(29)(31)	9.77E-03	2.61E-1
	3.3	E	EPC(15)(23)(32)	2.47E-02	2.48E-1
	3.4	C	EPC(2)(10)(15)(23)	5.12E-02	5.11E-1
	3.5	G	EPC(10)(26)(29)	5.97E-03	1.82E-1
	3.6	F	EPC(2)(8)(12)(24)	8.46E-02	6.12E-1
4	4.1	G	EPC(4)(15)	8.68E-03	3.19E-1
	4.2	E	EPC(2)(12)(17)	6.93E-02	5.74E-1
	4.3	F	EPC(5)(15)(10)	1.26E-02	2.33E-1
	4.4	F	EPC(4)(12)(13)	5.27E-02	3.77E-1
	4.5	G	EPC(6)(12)(15)	5.88E-04	2.89E-1
	4.6	G	EPC(7)(8)(11)	2.38E-04	3.27E-1
	4.7	G	EPC(8)(9)(12)	1.33E-04	2.82E-1
	4.8	C	EPC(10)(11)(15)	1.39E-01	7.83E-1
	4.9	E	EPC(7)(8)(20)	3.52E-02	4.79E-1
	4.10	E	EPC(10)(15)	7.17E-02	6.33E-1
	4.11	G	EPC(8)(13)(20)(21)	8.19E-04	3.21E-1
	4.12	D	EPC(7)(10)(12)(15)	9.37E-02	8.28E-1
	4.13	G	EPC(8)(10)(13)	6.88E-03	2.88E-1
	4.14	G	EPC(17)(18)(22)	4.39E-04	2.37E-1
	4.15	G	EPC(2)(4)	2.84E-03	3.99E-1

## Results and discussion

### Comparison with the original HEART approach

In this section, the IAHP-HEART method is compared with the original HEART approach to verify its feasibility and reasonableness in aviation safety assessment. Subtask 1.4 is taken as an example. The final HEP of subtask 1.4 calculated by the HEART and IAHP-HEART is illustrated in Table 4. In the original HEART method, the APOA of EPCs influencing human performance for subtask 1.4 is assessed by only an expert. The APOA of EPCs is the proportion of the effect relative to itself, and it refers to the absolute weight of EPCs. The final HEP based on the original HEART is obviously larger than the HEP based on the IAHP-HEART. The HEART result is subjective and unconvincing. However, the APOA calculation of EPCs in the proposed method is derived by the objective IAHP method. It represents the relative weight of EPCs, which largely reduces the subjectivity and uncertainty of experts’ judgment.

**Table 4:** Assessed proportion of affect (APOA) and human error probability (HEP) of subtask 1.4 based on different methods.

Subtask 1.4	APOA					HEP
HEART	0.6	0.7	0.4	0.2	0.2	4.85E-01
IAHP-HEART	0.2140	0.2389	0.1857	0.1776	0.1838	1.77E-02

HEART, human error assessment and reduction technique; IAHP, improved analytic hierarchy process.

Table 5 shows the mean HEP in different flight phases based on the HEART and IAHP-HEART. According to the IAHP-HEART method, the highest mean HEP is in the approach phase, and the second-highest mean HEP is in the landing phase. These two values are very close to each other. In contrast, according to the original HEART, the highest mean HEP appears in the landing phase, and the second-highest mean HEP is in the descending phase. The aviation safety data based on the Aviation Safety Reporting System managed by the National Aeronautics and Space Administration (NASA) are introduced to support the proposed method. Aviation incident data related to human factors between 2000 and 2019 are selected [41]. The numbers of incidents in the four flight phases are 3539, 5604, 10581, 7889. Then, the incident proportions connected with human factors can be expressed as 12.8%, 20.3%, 38.3%, and 28.6%. These data show that the highest HEP occurs in the approach phase and the second-highest HEP appears in the landing phase, followed by descent, climbing and cruise, in sequence. The results based on IAHP-HEART are consistent with the above actual data. Moreover, the total mean HEP values assessed by the HEART and IAHP-HEART are 3.88E-01 and 2.49E-02, respectively. Sandia National Laboratories (SNL) studied human error in a sophisticated man-machine system, and the results showed that the probability of human error for operating in the air is approximately 2.00E-2 [42]. The total mean HEP obtained by the proposed method is very close to that of SNL. This indicates that the results based on IAHP-HEART are rational and objective. Therefore, the application of the IAHP-HEART method to aviation flight safety assessment should be effective and feasible.

**Table 5:** Mean human error probability (HEP) in different flight phases.

Method	Mean HEP in different flight phases				Total mean HEP
	Climbing and cruise	Descent	Approach	Landing	
HEART	3.17E-01	3.92E-01	3.49E-01	4.18E-01	3.88E-1
IAHP-HEART	7.57E-03	1.42E-02	3.34E-02	3.30E-02	2.49E-02
Human factor event proportion	12.8%	20.3%	38.3%	28.6%	

HEART, human error assessment and reduction technique; IAHP, improved analytic hierarchy process.

In addition, it is remarkable that the HEP values for 9 of the 34 subtasks are higher than the average HEP value. In particular, the increase in HEP values in the landing process demonstrates that the operational performance of the flight crew may be influenced by mental overload or inattention. Furthermore, the highest HEP value appears in subtask 4.8 due to a lack of cognition and monitoring and incorrect manipulation. If the aircraft flaps are not laid down in time, the descent rate may increase, which may lead to deviation from the course. The HEP value of subtask 4.12 is the second highest value in all subtasks of the critical flight process. The “LANDING CHECKLIST” is an important guiding document that a flight crew can rely on during the critical flight stage, and it is also the last guarantee of flight safety. During the flight process, it guides the operational actions for the flight crew in a reasonable order, which makes the landing manipulation satisfy the inherent logic requirements of the aircraft system. Furthermore, the checklist can prevent the flight crew from missing critical actions and making mistakes and can provide quick action plans for the crew in emergency situations. The flight crew can easily make mistakes due to time constraints and low situational awareness.

The HEP value of subtask 2.8 is slightly smaller than that of subtask 3.6, and both have a high HEP in the whole flight process. The main reason is that the crew needs to pay more attention to controlling the aircraft in a short time according to the checklist. Specifically, the increase in mental and physical workload in the approach stage can augment the probability of human error. Moreover, the HEP values of subtask 4.2 and subtask 4.10 are relatively high. Although the two subtasks are at different stages of the landing process, the actions performed are similar, and the HEP values of the two subtasks are fairly close. The copilot is responsible for monitoring the speed and setting the flaps as commanded during the landing stage, but he/she may sometimes forget or ignore these tasks due to time constraints. Insufficient monitoring by the copilot may be a major factor contributing to this mistake. Three subtasks (3.4, 4.4 and 4.9) also have a high probability of human error in the whole flight process since these values are greater than the average HEP.

### Effect of EPCs on HEP

**Fig 3:**
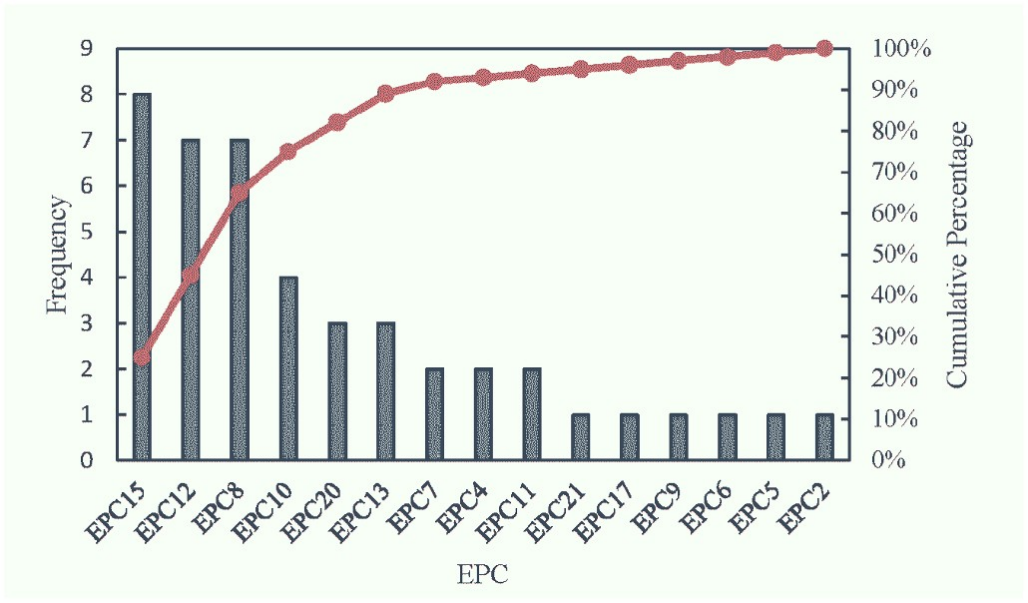
Critical error-producing condition (EPC) rankings based on the Pareto principle.

The Pareto principle is introduced to determine the pivotal EPCs in the landing process. This principle states that the most important variables are only approximately 20% in any industry [43]. In this paper, it can be used to classify and rank hazard factors that have a significant influence on flight safety. The statistical results, illustrated in Fig 3, can offer reasonable recommendations regarding the key EPCs to address in order to improve human performance reliability and enhance flight safety.

Fig 3 shows the hazard priority ranking of EPCs. According to the Pareto principle, the cumulative percentage of EPC15, EPC12, EPC8 and EPC10 is approximately 78%. The results indicate that the key EPCs influencing HEP are inexperience, channel overload, misperception of risk and knowledge transfer. Airlines or engineers should focus on taking corresponding remedial measures to improve these four EPCs. The inexperience EPC is ranked first among all EPCs. Management defects of airlines and inadequate training may be important factors leading to a pilot’s inexperience. On the one hand, airlines should strengthen crew resource management and establish efficient supervision institutions. On the other hand, pilots should receive more flight training, and airlines must regularly assess pilots’ skills. Channel overload is also a key factor increasing the error rate. During the landing phase, the pilot needs to perform multiple operational tasks in parallel, which may lead to channel conflicts, including visual, auditory and tactile information. Taking measures such as using a human-centered interface, performing balanced man-machine function allocation and utilizing an intelligent decision support system may contribute to reducing the pilot’s channel overload and error probability. The misperception of risk is the third key factor influencing human error. It is mostly derived from a decrease in situational awareness. Making flight tasks more interesting and improving human-computer interaction modes may be reasonable recommendations for maintaining high situational awareness. Finally, although it is difficult to ensure that pilots transfer specific knowledge from task to task without loss, pilots should consciously continue to learn relevant flight knowledge and accumulate more experience to reduce human errors.

## Conclusions

It is imperative to quantify human reliability in the aircraft manipulation process to minimize the occurrence of human errors. This paper proposes and applies an integrated human reliability quantitative method to assess flight safety in civil aviation. Although the original HEART method is an available tool for deriving HEP, it does not provide a concrete approach for analysts to calculate the APOA. This paper employs the IAHP method to determine the APOA value of each EPC. The IAHP method uses a fuzzy congruous matrix to replace the fuzzy judgment matrix, which can simplify the consistency test and ensure its accuracy compared with the AHP method. Then, these APOA values are integrated into the HEART approach to derive the HEP of flight tasks. The developed method can assist analysts in precisely calculating the APOA, and the HEP can be easily used to assess flight safety. In addition, a specific flight task based on the standard operation procedure is used in a case study. The results indicate that the proposed IAHP-HEART method is a reasonable and feasible evaluation tool in aviation safety assessment. Furthermore, it can be used for the evaluation, trade-off, and optimization of the flight operation process, and it provides a scientific theoretical decision basis for the design and development of a cockpit.

In future studies, larger amounts of human error data under various flight conditions should be collected and organized to improve variability and reduce uncertainty. Moreover, according to the framework of this paper and the human error database for flight accidents, user-friendly software can be designed to predict the HEP and operational risk in the critical flight process.

## Supporting information

S1 Table Descriptions of EPCs. (DOC)
