# Supplementary material for: Flight safety assessment based on an integrated human reliability quantification approach
Source: PLoS One. 2020 Apr 16;15(4):e0231391. doi: 10.1371/journal.pone.0231391 (PMC7161990; doi:10.1371/journal.pone.0231391)
Supplement: S1 Table — (DOC) [file pone.0231391.s001.doc]

**S1 Table.** Descriptions of EPCs

| No. | EPC | Effect of EPC | No. | EPC | Effect of EPC |
| --- | --- | --- | --- | --- | --- |
| 1 | Unfamiliarity | 17 | 20 | Educational mismatch | 2 |
| 2 | Time shortage | 11 | 21 | Dangerous incentives | 2 |
| 3 | Low signal-to-noise ratio | 10 | 22 | Lack of exercise | 1.8 |
| 4 | Feature override allowed | 9 | 23 | Unreliable instruments | 1.6 |
| 5 | Spatial and functional incompatibility | 8 | 24 | Absolute judgments required | 1.6 |
| 6 | Model mismatch | 8 | 25 | Unclear allocation of function | 1.6 |
| 7 | Irreversibility | 8 | 26 | Lack of progress tracking | 1.4 |
| 8 | Channel overload | 6 | 27 | Physical capabilities | 1.4 |
| 9 | Technique unlearning | 6 | 28 | Low meaning | 1.4 |
| 10 | Knowledge transfer | 5 | 29 | Emotional stress | 1.3 |
| 11 | Performance ambiguity | 5 | 30 | Poor health | 1.2 |
| 12 | Misperception of risk | 4 | 31 | Low morale | 1.2 |
| 13 | Poor feedback | 4 | 32 | Inconsistency of displays | 1.2 |
| 14 | Delayed/incomplete feedback | 4 | 33 | Poor environment | 1.15 |
| 15 | Inexperience | 3 | 34 | Low loading | 1.1 |
| 16 | Lack of information | 3 | 35 | Sleep cycle disruption | 1.1 |
| 17 | Inadequate checking | 3 | 36 | Task pacing | 1.06 |
| 18 | Objective conflict | 2.5 | 37 | Supernumeraries | 1.03 |
| 19 | No diversity | 2.5 | 38 | Age | 1.02 |
